# MicroRNA-Mediated Regulatory Networks in *Helicobacter pylori*–Associated Gastric Cancer

**DOI:** 10.1155/bri/6662299

**Published:** 2025-11-26

**Authors:** Omid Karimdadi Sariani, Mahroo Mohamadi, Zahra Gharagheizi, Mohammad Alaee, Masoumeh Beig, Mohammad Sholeh

**Affiliations:** ^1^Department of Genetics, College of Sciences, Kazerun Branch, Islamic Azad University, Kazerun, Iran; ^2^Department of Medical Laboratory Sciences, School of Allied Medical Sciences, Shahid Beheshti University of Medical Sciences, Tehran, Iran; ^3^Department of Biology, Science and Research Branch, Islamic Azad University, Tehran, Iran; ^4^Metabolic Disorders Research Center, Endocrinology and Metabolism Molecular-Cellular Sciences Institute, Tehran University of Medical Sciences, Tehran, Iran; ^5^Department of Bacteriology, Pasteur Institute of Iran, Tehran, Iran

**Keywords:** gastric cancer, *Helicobacter pylori*, inflammation, microRNAs, miRNA

## Abstract

Gastric cancer (GC) remains a complex disorder with an unclear etiology. *Helicobacter pylori*, a Gram-negative bacterium, plays a key role in the development of chronic gastritis, peptic ulcers, anemia, and GC. It survives and proliferates in macrophage autophagosomes, gastric epithelial cells, and dendritic cells, with its cytotoxin-associated gene A (*CagA*) and vacuolating Toxin A (*VacA*) interfering with autophagy. While most infected patients develop chronic gastritis, 10%–15% develop ulcers, and 1%–3% progress to GC. MicroRNAs (miRNAs), small noncoding RNAs, regulate genes by modulating tumor suppressors and oncogenes, and are crucial in GC. This review explores miRNAs as potential biomarkers and therapeutic targets for early GC diagnosis, monitoring progression, and predicting treatment outcomes. Dysregulated miRNAs such as miR-124, miR-145, and miR-21 have been identified in GC. Additionally, miRNAs influence *H. pylori*-induced inflammation and the expression of virulence factors. Targeting miRNAs could reduce *H. pylori*-related diseases, including gastritis, ulcers, and GC. However, further research is required to fully understand miRNAs' role in *H. pylori* infection and develop miRNA-based therapies.

## 1. Background on *Helicobacter pylori* Infection and Gastric Cancer (GC)

*H. pylori* is a Gram-negative, microaerophilic bacterium. This causes chronic gastritis, peptic ulcers, iron deficiency anemia, gastric mucosa–associated lymphoid tissue lymphoma, and GC [1]. Phylogenetic comparisons of the human and *H. pylori* lineages have suggested a single host switch [2–4]. High-frequency recombination and mutations in *H. pylori* during human life have led to its adaptation to human biological features [5]. Although *H. pylori* has been detected in cats, suggesting that it could be transferred from pets to humans, it appears to be the main reservoir for infected populations [6]. Health, poverty, population congestion, water pollution, and individual habits influence the prevalence of *H. pylori* infections [7, 8]. In underdeveloped countries, *H. pylori* prevalence is 85%–95%; in developed countries, 30%–50%; and reaches over 90% during adulthood in developing countries [4, 8, 9]. According to a systematic review and meta-analysis, *H. pylori* recurrence has increased over the last 10 years and remains a global public health challenge. The *H. pylori* recurrence rate increases with time after eradication and varies according to region, sex, and method [10]. *H. pylori* is a Type I carcinogen, according to the World Health Organization.

### 1.1. GC

More than one million cases of GC are estimated to be diagnosed in 2020, with considerable geographic variation. Eastern Asian and Eastern European regions showed the highest incidence rates; globally, approximately two-thirds of all cases occur in men [11]. Incidence and mortality are correlated, reflecting poor survival and highlighting the importance of preventing cases or earlier diagnosis. GC is the fifth most commonly diagnosed cancer and the third leading cause of cancer-related deaths worldwide [12], accounting for approximately 1.1 million new cases and 770,000 deaths in 2020, and colorectal cancer (CRC) is ranked second in cancer-related deaths [13]. If current trends continue, the global incidence will reach an estimated 1.77 million new cases by 2040 [13]. East Asia, particularly countries such as Japan, South Korea, and China, has the highest burden of GC, accounting for approximately 75% of new cases worldwide [14]. GC incidence remains notably high in both Japanese and Korean populations [15]. In addition to the different prevalences of East Asian-type cytotoxin-associated Gene A (*cagA*) strains and Western-type CagA strains of *H. pylori*, cultural and dietary factors may also be significant [16–18]. In 2020, stomach cancer ranked as the fifth most frequently diagnosed malignancy worldwide, with an estimated 1.1 million new cases, and was the fourth leading cause of cancer-related deaths, accounting for approximately 800,000 fatalities [19].

Intestinal GC is typically associated with environmental variables, whereas diffuse GC is associated with host genetic factors [20].

Although *H. pylori* infection significantly increases the risk of developing diffuse GC and intestinal-type gastric adenocarcinoma, chronic inflammation is not required to create diffuse-type cancers. This suggests that the mechanisms underlying the ability of *H. pylori* to induce malignancy differ among cancer subtypes [21]. Diffuse-type carcinoma cells secrete mucus into the interstitium. Cells with large amounts of mucin in the cytoplasm displace the nucleus to the cell periphery (signet-ring cell carcinoma) [22, 23].

Recent research indicates that cells produced from the bone marrow (gastric stem cells) contribute to tumor formation [24]. Patients with excessive acid secretion are predisposed to develop duodenal ulcers if they have antral-predominant gastritis. Low acid secretion increases the likelihood of developing gastritis, gastric ulcer, gastric atrophy, intestinal metaplasia, and dysplasia, and in rare cases, GC is particularly prevalent among older individuals. *H. pylori* infection leads to MALT lymphoma of the stomach mucosa [25].

### 1.2. *H. pylori* Virulence Factors in Gastric Carcinogenesis

*H. pylori* infection plays a significant role in gastric carcinogenesis, with clinical outcomes influenced by bacterial, host genetic, and immunological factors. Chronic gastritis develops in 90% of infected individuals, often without symptoms [26]. The bacterium survives the stomach's acidic environment by secreting urease, neutralizing stomach acid, and using flagellar motility to reach gastric epithelial cells [27]. Adhesion factors such as blood group antigen-binding adhesin (BabA) and sialic acid–binding adherence (SabA) facilitate attachment to the gastric surface, where *H. pylori* forms microcolonies and accesses nutrients such as iron [28].

**Figure 1:**
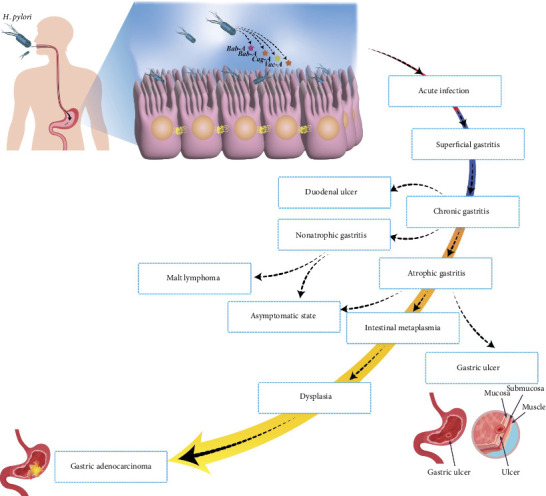
The progression of gastric diseases and conditions associated with *Helicobacter pylori* infection. The pathway begins with acute infection in the gastric mucosa, leading to different stages such as superficial gastritis, chronic gastritis, and gastric ulcers. In some cases, it can evolve into more severe conditions such as duodenal ulcers, nonatrophic gastritis, atrophic gastritis, and intestinal metaplasia. Additionally, the infection can lead to malt lymphoma, dysplasia, and eventually gastric adenocarcinoma. The image also highlights the role of specific *H. pylori* virulence factors (*BabA*, *CagA*, and *VacA*) in disease progression. BabA: blood group antigen–binding adhesin, CagA: cytotoxin-associated Gene A, and VacA: vacuolating Toxin A.

The CagA oncoprotein, injected via the Type IV secretion system (T4SS), is crucial in GC development. CagA interacts with host cell proteins through phosphorylation, promoting cytoskeletal changes, cell–cell junction breakdown, and increased cell proliferation [29–31]. VacA, another key toxin, disrupts epithelial cells and modulates the immune response, contributing to chronic inflammation and carcinogenesis [32, 33]. CagA and VacA synergistically interfere with autophagy, allowing CagA to accumulate within cells and further promote oncogenesis [34] (Figure 1).

*H. pylori* infection triggers a strong immune response, releasing antimicrobial peptides and inflammatory cytokines such as IL-8 and IL-6 [35, 36]. This chronic inflammation leads to DNA mutations, inhibits apoptosis, and stimulates angiogenesis and cell proliferation—key steps in the development of GC. Additionally, *H. pylori* induce oxidative stress and autophagy, creating an environment conducive to carcinogenesis. The HP0175 antigen activates autophagy through the PERK/ATF4/CHOP pathway, further enhancing the persistence of *H. pylori* and increasing GC risk [37].

### 1.3. *H. pylori-*Induced Epigenetic Changes

Changes that affect gene expression but not DNA sequences are epigenetic. Molecular studies have shown that GC progression is affected by environmental and genetic factors. Notably, these modifications are crucial for carcinogenesis [38]. *H. pylori* infection promotes DNA damage by creating ROS, resulting in epigenetic alterations that may contribute to GC [39]. Double-strand DNA breaks are caused by *H. pylori* infection of the epithelial cells. A recent study demonstrated the role of histone modification in GC [40]. The core units are H3 and H4 histones with extended tails outside the nucleosomes. Adenosine diphosphate ribosylation and ubiquitination are amino acid sequence modifications. These chromatin structural modifications enable regulatory proteins to control transcription by acquiring DNA as a silent switch. These histone modifications are crucial for gene regulation, DNA repair, and cell proliferation, and their dysregulation can lead to cancer [40, 41]. Epigenetic alterations can trigger cancer development. DNA methylation, which causes the spread of *H. pylori* infection and inflammation, is the most significant modification followed by DNA methylation disorders. Mutations and epigenetic alterations cause neoplasia [41]. The infection of epithelial cells with *H. pylori* induces apoptosis, proliferation, and differentiation, ultimately forming oncogenic epithelial cells. Free radicals methylate DNA cytosine, and NO can boost DNA methyltransferase activity [42]. The inflammation and signaling pathways induced by *H. pylori* infection are likely to contribute to the carcinogenicity of the stomach. Immunological responses, apoptosis, cell cycle, autophagy, and other events alter microRNA (miRNA) expression [40, 43].

## 2. Overview of miRNAs

miRNAs are a class of small, noncoding RNA molecules that are approximately 22 nucleotides in length [44]. miRNAs involve various biological processes, including cell proliferation, differentiation, apoptosis, and metabolism [45]. miRNAs regulate gene expression by binding to target mRNAs' UTR and either promoting or repressing their translation [44]. miRNAs are produced from long RNA transcripts called pri-miRNAs that RNA Polymerase II transcribes [46]. Pri-miRNAs are then processed by the Drosha and Dicer enzymes to form mature miRNAs [47]. Mature miRNAs are then exported to the cytoplasm, where they can bind to target mRNAs and regulate their expression [48]. In 2024, Victor Ambros and Gary Ruvkun were awarded the Nobel Prize in Physiology or Medicine for their pioneering discovery of miRNAs, a class of small noncoding RNAs that play a key role in regulating gene expression. This discovery dates back to the early 1990s [49, 50] and marked a significant advancement in molecular biology.

Small, evolutionarily conserved molecules, including transcription factors, RNA-binding proteins, and small noncoding RNAs, regulate the expression of miRNAs. miRNAs are involved in various biological processes, such as proliferation, apoptosis, cell differentiation, metabolism, and epithelial–mesenchymal transition, and, unsurprisingly, miRNAs are implicated in carcinogenesis [51, 52]. Thus, miRNAs are considered new immune regulators. miRNAs are stable in patient sera and can be used as early markers of bacterial infections because they are not readily degraded [36, 53]. Studies of miRNAs have provided a better understanding of cellular biology and immunology [54]. Therefore, new studies are beginning to analyze how miRNAs act as tumor suppressors or oncogenic miRNAs to promote tumor development.

Dysregulated miRNAs can act as biomarkers of *H. pylori* infection and GC. miRNAs can also serve as therapeutic targets for *H. pylori* infection and GC [55].

### 2.1. miRNAs as Biomarkers for GC

Recent research has highlighted the growing potential of miRNAs as biomarkers for GC [56]. Dysregulated miRNAs are increasingly recognized for their ability to detect, diagnose, monitor, and predict treatment responses in GC [57].

These small noncoding RNAs serve as indicators of disease progression and treatment efficacy, and their presence in easily accessible body fluids makes them highly promising for noninvasive diagnostics.

Among the most studied miRNAs in GC, miR-21 and miR-106b-25 have demonstrated significant potential as biomarkers [58]. Elevated levels of miR-21 have been linked to a poor prognosis, with overexpression correlating with advanced disease stages and metastasis [59]. A growing body of clinical studies has focused on validating the diagnostic and prognostic utility of miRNAs in GC. For instance, the authors in [60] demonstrated that miRNA profiles could differentiate between GC patients and healthy controls with a sensitivity of 90% and specificity of 95%. This highlights the strong diagnostic potential of miRNAs, especially when used in conjunction with other biomarkers or imaging techniques. Furthermore, miRNAs have shown promise in predicting recurrence risk, with sensitivity and specificity rates of 80% and 90%, respectively. Such results suggest that miRNAs could be integrated into clinical practice for more accurate risk stratification and personalized treatment strategies.

In addition to diagnosis and prognosis, miRNAs are also being studied for their role in monitoring treatment responses. Research by Angerilli et al. [61] found that miRNA expression profiles could help predict which patients would respond to specific therapies and identify those at risk of treatment resistance. This ability to guide therapeutic decisions is crucial for improving patient outcomes and minimizing unnecessary treatments [61]. While the study of miRNAs as biomarkers for GC is rapidly advancing, challenges remain in terms of clinical validation and widespread implementation. The variability in miRNA expression across populations and cancer subtypes, as well as the need for standardized methodologies in miRNA detection, are significant barriers that require attention. However, with ongoing technological advancements and more robust clinical trials, miRNAs hold great promise in revolutionizing the early detection, monitoring, and management of GC.

### 2.2. miRNAs as Potential Therapeutic Targets

Studies have shown that miRNAs can regulate *H. pylori*-induced inflammation and immune response. For example, a study revealed that miR-155 may regulate the inflammatory response to *H. pylori* infection [62]. Additionally, miR-146a regulates the expression of Interleukin 1 receptor-associated Kinase 1, a key mediator of *H. pylori*-induced inflammation [62].

Furthermore, miRNAs have been shown to regulate *H. pylori* virulence factors such as CagA and VacA, which are associated with an increased risk of GC. For example, miR-584 targets CagA and reduces its expression [63]. Targeting miRNAs that regulate *H. pylori*-induced inflammation and pathogenic factors may reduce *H. pylori*-induced inflammation and related diseases [64]. However, limited evidence supports the feasibility of using miRNAs as viable therapeutic targets. Further research is required to fully understand the role of miRNAs in *H. pylori* infection, which is associated with GC, and develop effective miRNAs as potential therapeutic targets.

### 2.3. Impact of *H. pylori* Inflammatory Factors on miRNAs

*H. pylori* infection increases the risk of GC through inflammation and the abnormal development of stomach cells. Nod-like receptor NACHT, LRR, and PYD domain-containing Protein 3 (NLRP3), an inflammasome component, triggers chronic inflammation. Li et al. discovered that NLRP3 overexpression activates inflammasomes and promotes the growth of macrophages and GC cells. Moreover, NLRP3 binds to the cyclin D1 promoter, inducing its expression in GC cells and promoting growth and carcinogenesis [65]. In *H. pylori* infection, miR-22 levels decrease, whereas NLRP3 levels increase, promoting the unrestricted growth of gastric epithelial cells and GC development [66]. Additionally, miR-223-3p levels are elevated during *H. pylori* infection and target ARID1A, a cell growth and differentiation protein. The binding-mediated inhibition of ARID1A expression is essential in cellular behavior, particularly concerning the facilitation of proliferation and metastasis in cells reliant on the CagA signaling pathways [67]. Autophagy has been shown to inhibit tumor growth. Yang et al. discovered an increased expression of miR-99b in *H. pylori*-positive GC tissues and GC cells. Overexpression of miR-99b in cells had effects on cell proliferation and autophagy. miR-99b suppresses mTOR to initiate its effects on autophagy, suggesting that it may be a potential therapeutic target for gastrointestinal diseases caused by *H. pylori* [55]. *H. pylori* infection elicits upregulation of specific miRNAs, such as miR-146a, miR-223-3p, and miR-1289. m-146a is induced by *H. pylori* infection in an NF-κB-dependent manner, independent of the proinflammatory cytokines IL-8, IL-1b, and TNF-α. Similarly, miR-1289 is overexpressed upon *H. pylori* infection and directly interacts with the 3′-UTR of the H-K-ATPase subunit protein [68, 69]. Yang et al.'s findings underscore that miR-223-3p is activated by *H. pylori* CagA and necessitates NF-κB for its transcription from the promoter region. miR-223-3p, an oncogenic miRNA, has been implicated in diverse cancer types, including GC [68, 70]. miR-370 has been identified as a direct modulator of Forkhead box M1mRNA, which is upregulated by CagA, thereby influencing gastric cell growth and contributing to the pathogenesis of gastritis and GC [71]. Suppression of *H. pylori* has been associated with decreased methylation and elevated miR-133a expression [68, 72]. miR-222, implicated in various cancer types, including CRC, glioma, lymphoma, melanoma, hepatocellular carcinoma, and lung, breast, and thyroid cancers, has been investigated in the context of GC [73]. Although GC tissue samples exhibit increased miR-222 expression, discernible differences between *H. pylori*-positive and -negative patients remain unclear. Further validation is warranted to establish the potential contribution of miR-222 upregulation to GC development.

### 2.4. Role of miRNAs in GC Associated With *H. pylori* Infection

**Figure 2:**
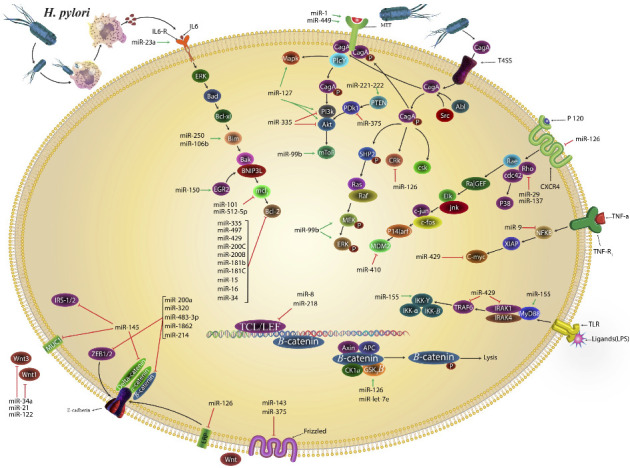
A brief overview of the changes in miRNAs due to *Helicobacter pylori* infection and the proteins and genes targeted by these miRNAs. The figure illustrates the molecular interactions between *Helicobacter pylori* infection and host cell signaling pathways that contribute to the development of gastric cancer. *H. pylori* virulence factors, such as CagA and VacA, interfere with normal cellular processes by disrupting key intracellular signaling cascades. This disruption affects a range of microRNAs, including miR-21, miR-34a, miR-145, miR-375, miR-429, and miR-99b, which in turn regulate critical processes such as cell proliferation, apoptosis, autophagy, and inflammation. The Wnt/β-catenin pathway is one of the key pathways impacted, with miRNAs such as miR-34a and miR-21 downregulating tumor suppressors such as E-cadherin, leading to the accumulation of β-catenin and promoting oncogenesis. Another significant pathway affected is the PI3K/Akt/mTOR pathway, regulated by miR-99b and miR-335, which influence cell survival and proliferation. Downregulation of these miRNAs leads to unchecked cell growth and cancer progression. Additionally, the MAPK pathway is influenced by *H. pylori*'s CagA protein, with miRNAs such as miR-21 and miR-222 promoting oncogenic signaling through Ras-Raf-MEK-ERK, driving increased cell proliferation. *H. pylori* also impact the NF-κB pathway, with miRNAs such as miR-155 and miR-429 modulating NF-κB, a key regulator of inflammation. Activation of NF-κB by *H. pylori* leads to the production of inflammatory cytokines, contributing to chronic inflammation and cancer progression. Apoptosis regulation is another critical aspect affected by miRNAs such as miR-145 and miR-15/16, which target antiapoptotic proteins such as Bcl-2 and Mcl-1. These proteins are regulated by *H. pylori* virulence factors, particularly CagA. Dysregulation of these miRNAs results in decreased apoptosis and enhanced cell survival, promoting oncogenesis. Overall, the interplay between *H. pylori*, miRNAs, and these key signaling pathways demonstrates the central role of miRNAs as both tumor suppressors and oncogenes in gastric cancer. miRNAs such as miR-21, miR-34a, miR-145, and miR-375 emerge as potential biomarkers for early detection and therapeutic targets to control the progression of *H. pylori*-induced gastric cancer.

Several miRNAs play crucial roles in the intricate interplay between *H. pylori* infection and GC, each with its associated mechanisms or relationships with *H. pylori*. Notably, miR-584 and miR-1290 are elevated in CagA-transformed cells, impacting let-7 expression through CagA-derived epigenetic regulation [70]. Additionally, miR-203, miR-204, miR-375, and miR-27b are significantly overexpressed in *H. pylori*-infected tissues, contributing to neoplastic transformation and invasion [74, 75]. miR-218 exhibits lower expression in *H. pylori*-positive gastric biopsies [76]. Other miRNAs, including miR-146, miR-21, miR-1289, miR-223-3p, and miR-99b, were significantly upregulated in *H. pylori*-positive GC tissues and BGC-823 GC cells [77]. miR-122, with elevated plasma levels in GC, potentially serves as an indicator of *H. pylori* infection. miR-320 is inhibited by *H. pylori*, leading to increased levels of Mcl-1 and suppressed apoptosis. miR-483 engages in biological pathways crucial for *H. pylori* infection and GC development, whereas miR-622 is associated with *H. pylori* infection and has a regulatory impact on broader cellular processes. miR-629 is downregulated and correlated with GC lymph node metastasis, suggesting its potential modulation by *H. pylori* [78]. miR-672 is downregulated by *H. pylori*, contributing to the microbial influence on miRNA dysregulation [79]. Finally, miR-129-2, epigenetically re-expressed in GC, leads to decreased Sox4 expression, establishing a link between *H. pylori* infection and epigenetic regulation of miRNAs in cancer development [80]. Collectively, these miRNAs contribute to the complex molecular landscape of GC in the context of *H. pylori* infection, influencing various cellular processes, such as proliferation, migration, apoptosis, and drug resistance. Understanding these miRNA alterations provides insights into the potential diagnostic and therapeutic strategies for managing *H. pylori*-related GC. A systematic overview in Table 1 catalogs the involvement of miRNAs in GC, outlining their roles in cell proliferation, migration, apoptosis, and drug resistance.  Figure 2 provides an overview of the miRNAs dysregulated by *H. pylori* infection and highlights their corresponding molecular targets, illustrating the complex regulatory network underlying *H. pylori*-induced gastric carcinogenesis.

## 3. Conclusion

In conclusion, this study unveils the intricate interplay between miRNAs, *H. pylori* infection, and GC, providing insights into the complex molecular dynamics that drive GC development. Dysregulated miRNA expression, influenced by *H. pylori*-associated factors, such as CagA and VacA, plays a central role in modulating crucial cellular processes. Altered expression of specific miRNAs, such as miR-155, miR-146a, miR-218, and miR-21, reflects the dynamic interplay between microbial factors and host response. miRNAs serve as promising biomarkers for GC detection, diagnosis, and monitoring and have therapeutic potential for alleviating *H. pylori*-induced inflammation. Changes in miRNAs, such as miR-584 and miR-1290, highlight their roles in driving cellular transformations linked to *H. pylori* infection. Although eradicating *H. pylori* improves miRNA dysregulation in the stomach, challenges persist in addressing miRNA alterations in intestinal metaplasia. Understanding the specific miRNA landscape associated with *H. pylori* in GC establishes these molecules as key players and potential therapeutic targets. A deeper understanding of the miRNA regulatory network in GC promises novel diagnostic and therapeutic strategies, advancing our understanding of the molecular intricacies of *H. pylori*-associated GC.

### 3.1. Future Directions

Currently, we are beginning to understand the connection between miRNAs and GC. miRNAs have a complex regulatory network, and the number of miRNA genes that have been identified is increasing. Future research will expand our knowledge of the effects of miRNAs in GC.

## Figures and Tables

**Table 1:** An overview of microRNAs in *H. pylori*-associated gastric cancer.

MicroRNA	Expression status	Target/function	Role in gastric cancer (GC)	Reference(s)
miR-10b	Upregulated	Predicts and treats gastric cancer by increasing levels	Increases gastric cancer progression	[81]
miR-23a	Downregulated	Inhibits IL6-R production, promoting gastric adenocarcinoma growth	Promotes growth of gastric adenocarcinoma	[82]
miR-31/32	Upregulated	Expression significantly higher in gastric cancer tissue	Associated with gastric cancer tissue growth	[83]
miR-43c	Downregulated	Targets VEZT protein, inhibiting its production	Inhibits gastric cancer metastasis	[84]
miR-141	Varied	Contributes to gastric cancer progression by influencing individual cell growth	Modulates cell growth in gastric cancer	[85]
miR-192	Upregulated	Overexpression associated with migration of gastric cancer cells	Promotes cell migration in gastric cancer	[86]
miR-194	Downregulated	Suppresses EMT in gastric cancer cells, preventing cell migration and invasion	Suppresses metastasis	[87]
miR-218	Downregulated	Inhibits the growth of gastric cancer	Inhibits gastric cancer growth	[88]
miR-222	Varied	Suggested for treating gastric cancer	Potential therapeutic target for gastric cancer	[89]
miR-335	Downregulated	Inhibits gastric cancer progression and spread	Suppresses metastasis and tumor spread	[90]
miR-367	Downregulated	Inhibits invasion and metastasis of gastric cancer	Suppresses invasion and metastasis in gastric cancer	[91]
miR-424/503	Varied	Expression associated with lymph node metastasis	Associated with metastasis in gastric cancer	[92]
miR-518a-2	Upregulated	Associated with tumor size, lymph node metastasis, and local invasion	Associated with larger tumor size and metastasis	[93]
miR-15/16	Downregulated	Modulates apoptosis and targets BCL2, contributing to multidrug resistance	Contributes to resistance in gastric cancer	[94]
miR-27	Downregulated	Slows the growth of gastric cancer cells	Inhibits gastric cancer cell growth	[95]
miR-29 family	Downregulated	Inhibits proliferation and migration of gastric cancer cells	Reduces proliferation and migration of gastric cancer cells	[96]
miR-34	Upregulated	Restores p53 function in cells without p53	Enhances p53 activity, suppressing gastric cancer growth	[97]
miR-133a	Downregulated	Suppresses malignancies	Inhibits malignancy in gastric cancer	[89]
miR-143	Downregulated	Expression significantly decreased in gastric cancer tissues	Decreased expression leads to gastric cancer progression	[98]
miR-146a	Varied	Reduces inflammation by targeting IRAK1 and TRAF6	Functions as a feedback loop in gastric cancer	[99]
miR-148a	Varied	May inhibit the activity of its regulated PIN, acting as a tumor suppressor	Potential tumor suppressor role in gastric cancer	[100]
miR-149	Downregulated	Inhibits the growth of gastric cancer	Inhibits gastric cancer cell growth	[101]
miR-152	Varied	Reduces the proliferation and motility of gastric cancer cells	Impairs proliferation and motility in gastric cancer	[102]
miR-181b	Downregulated	Targets BCL2, helpful in treating multidrug resistance	Inhibits multidrug resistance in gastric cancer	[103]
miR-182	Downregulated	Inhibits the proliferation of gastric cancer cells and the formation of colony structures	Suppresses cell proliferation and colony formation	[104]
miR-196a	Upregulated	Overexpression associated with gastric cancer therapies	Associated with enhanced therapeutic responses in gastric cancer	[105]
miR-199a	Upregulated	Promotes gastric cancer cell proliferation and metastasis	Increases cell proliferation and metastasis in gastric cancer	[106]
miR-205	Varied	Plays a significant role in the progression of gastric cancer	Affects cancer progression with variable expression	[107]
miR-214	Upregulated	Overexpression associated with migration of gastric cancer cells	Facilitates migration of gastric cancer cells	[108]
miR-329	Varied	Identified as either a tumor suppressor or an oncogene	Acts variably as a tumor suppressor or oncogene	[109]
miR-331-3p	Upregulated	Potential therapeutic use in restoring gastric cancer cells	Potential therapy for restoring gastric cancer cells	[110]
miR-375	Downregulated	Downregulated in primary gastric cancers	Inhibits progression of primary gastric cancers	[111]
miR-410	Downregulated	Significantly lower in gastric cancer, associated with lymph node metastasis	Associated with lymph node metastasis in gastric cancer	[112]
miR-15/16	Downregulated	Modulates apoptosis and targets BCL2, contributing to multidrug resistance	Contributes to multidrug resistance in gastric cancer	[94]
miR-27	Downregulated	Slows the growth of gastric cancer cells	Inhibits gastric cancer cell growth	[95]
miR-106a	Upregulated	Upregulation causes gastric cancer metastasis	Promotes metastasis in gastric cancer	[113]
miR-107	Upregulated	Overexpression causes metastasis	Associated with metastasis in gastric cancer	[114]
miR-122	Downregulated	Decreased levels associated with improved early identification of DM in GC	Decreased expression aids early gastric cancer detection	[115]
miR-124a	Downregulated	Inhibits gastric cancer cell growth by targeting EZH2	Inhibits gastric cancer cell growth by targeting EZH2	[116]
miR-155	Upregulated	Targets the cAMP pathway, impacting cell growth	Enhances growth in gastric cancer cells	[117]
miR-181b	Downregulated	Targets BCL2, helpful in treating multidrug resistance	Helps in treating multidrug resistance in gastric cancer	[103]
miR-196a	Upregulated	Overexpression associated with gastric cancer therapies	Associated with therapeutic responses in gastric cancer treatments	[105]
miR-199a	Upregulated	Promotes gastric cancer cell proliferation and metastasis	Increases gastric cancer cell proliferation and metastasis	[106]
miR-221-222 cluster	Varied	Suggested for treating gastric cancer	Suggested as a therapeutic target for gastric cancer	[118]
miR-320	Downregulated	Inhibited by *H. pylori*, contributing to an increased risk of gastric cancer	Associated with an increased risk of gastric cancer when inhibited	[119]
miR-328	Downregulated	Suppression could increase CD44 expression, hastening cancer progression	Contributes to cancer progression when suppressed	[120]
miR-331-3p	Upregulated	Potential therapeutic use in restoring gastric cancer cells	Potentially restores gastric cancer cells as a therapy	[110]
miR-335	Downregulated	Inhibits gastric cancer progression and spread	Suppresses gastric cancer progression and metastasis	[90]
miR-379	Downregulated	Downregulated in cisplatin-resistant GC cell lines	Involved in resistance to cisplatin in gastric cancer cell lines	[121]

## Data Availability

The data that support the findings of this study are available in the supporting information of this article.
